# Therapeutic Targeting of Rab GTPases: Relevance for Alzheimer’s Disease

**DOI:** 10.3390/biomedicines10051141

**Published:** 2022-05-16

**Authors:** Kate L. Jordan, David J. Koss, Tiago F. Outeiro, Flaviano Giorgini

**Affiliations:** 1 Department of Genetics and Genome Biology, University of Leicester, University Road, Leicester LE1 7RH, UK; klj19@leicester.ac.uk; 2 Faculty of Medical Sciences, Translational and Clinical Research Institute, Newcastle University, Newcastle Upon Tyne NE2 4HH, UK; david.koss@newcastle.ac.uk (D.J.K.); tiago.outeiro@newcastle.ac.uk (T.F.O.); 3 Department of Experimental Neurodegeneration, Center for Biostructural Imaging of Neurodegeneration, University Medical Center Göttingen, 37075 Göttingen, Germany; 4 Max Planck Institute for Natural Sciences, 37075 Göttingen, Germany; 5 Scientific Employee with a Honorary Contract at Deutsches Zentrum für Neurodegenerative Erkrankungen (DZNE), 37075 Göttingen, Germany

**Keywords:** Rab GTPases, Alzheimer’s, neurodegeneration

## Abstract

Rab GTPases (Rabs) are small proteins that play crucial roles in vesicle transport and membrane trafficking. Owing to their widespread functions in several steps of vesicle trafficking, Rabs have been implicated in the pathogenesis of several disorders, including cancer, diabetes, and multiple neurodegenerative diseases. As treatments for neurodegenerative conditions are currently rather limited, the identification and validation of novel therapeutic targets, such as Rabs, is of great importance. This review summarises proof-of-concept studies, demonstrating that modulation of Rab GTPases in the context of Alzheimer’s disease (AD) can ameliorate disease-related phenotypes, and provides an overview of the current state of the art for the pharmacological targeting of Rabs. Finally, we also discuss the barriers and challenges of therapeutically targeting these small proteins in humans, especially in the context of AD.

## 1. Rab GTPases: Function and Biological Roles

**Figure 1:**
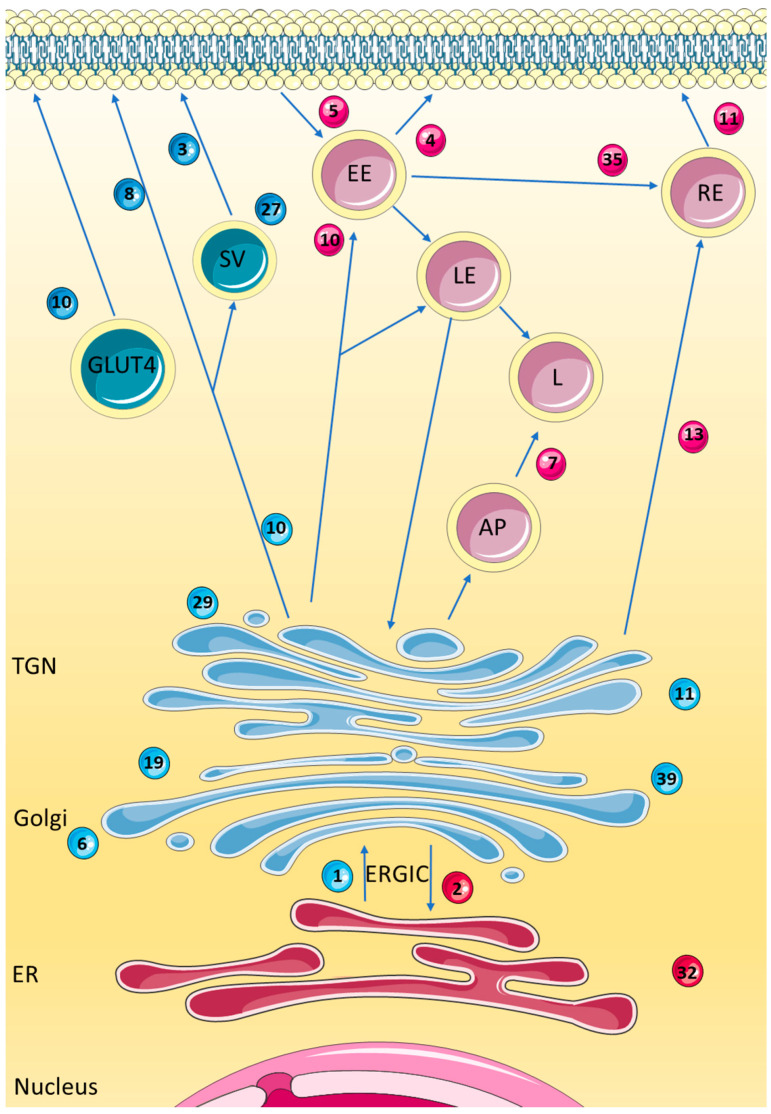
The Intracellular pathways and a selected number of Rab GTPases associated with the endoplasmic reticulum (ER), Golgi, trans-Golgi network (TGN), and endosomal pathways. Rab1 and Rab2 are localised to the ER and Golgi, and play a role in the ER to Golgi apparatus trafficking pathway, via the endoplasmic reticulum to Golgi intermediate compartment (ERGIC). Rab3 is localised to synaptic secretory vesicles (SV) and the plasma membrane and is involved in exocytosis and neurotransmitter release. Rab4 has a role in protein recycling and transport to the plasma membrane and is localised to early endosomes (EE). Rab5 is localised to the EE and aids its fusion and formation. Rab6 is involved with regulating intra-Golgi trafficking. Rab7 is localised to the late endosome (LE), lysosome (L), and autophagosomes (AP) and is involved in the maturation and transport between these vesicles. Rab8 is associated with exocytosis from the TGN to the plasma membrane, with localisation to the plasma membrane and SV. Rab10 is localised to the ER, Golgi, endosomes, and GLUT4 vesicles and is involved in ER dynamics, endocytosis, and trafficking to the plasma membrane. Rab11 is also localised to the Golgi, as well as the recycling endosome (RE) and EE. Rab13 is involved in the TGN and RE to plasma membrane transport pathway. Rab19 has been shown to localise to the Golgi, however there is little known about its role. Rab27 is involved in exocytosis, localising to SV. Rab29 and Rab39 are both localised to the Golgi. Rab32 localises to the ER and mitochondria, with a role in mitochondrial dynamics and autophagy. Rab35 localises to the plasma membrane, and is involved in endocytic recycling. Rabs more strongly associated with secretory pathways are shaded in blue while those more strongly associated with endosomal pathways are shown in red. Adapted with permission from Hutagalung et al. 2022, American Physiological Society [7].

Rab GTPases (Rabs) are a family of small proteins classified within the larger Ras superfamily of guanosine triphosphatase (GTP) binding proteins [1]. Rabs play key roles in vesicle trafficking between different cellular compartments, including the endoplasmic reticulum (ER), Golgi, endosomes, and plasma membrane (Figure 1). There are over 60 mammalian Rabs, with orthologs found in many different species, including *D. melanogaster, S. cerevisiae, A. thaliana,* and *C. elegans* [2,3,4,5]. Indeed, several yeast Rab GTPases can be functionally replaced by mammalian homologs as their functions are so highly conserved [6]. Many Rabs are ubiquitously expressed, although some are tissue specific [6].

**Figure 2:**
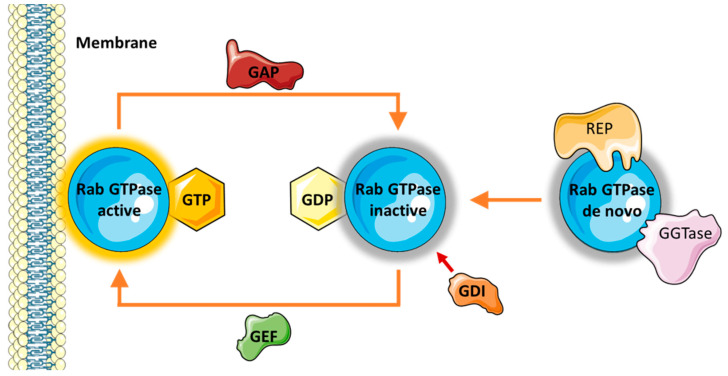
The cycle of Rab GTPase activation. Newly synthesised de novo Rab GTPases interact with Rab escort protein (REP), which enables prenylation via geranylgeranyltransferase (GGTase). When active, Rab GTPases are bound to GTP and associated with their target membrane. Following hydrolysis of GTP to GDP, they become inactive and reside in the cytosol. The Rab GTPase activation cycle is aided by a number of effectors. GTPase activating proteins (GAPs) catalyse the hydrolysis of GTP to GDP to inactivate the Rab. GDP dissociation inhibitors (GDIs) retrieve the inactive Rab from the membrane and solubilises it in the cytosol. However, guanine exchange factors (GEFs) catalyse the exchange of GDP with GTP, thus reactivating the Rab [12].

Rabs must be activated in order to function, which occurs through a cycle of GTP and GDP binding controlled by a number of different accessory proteins (Figure 2). Guanine exchange factors (GEFs) are responsible for recruiting the respective Rab to the target membrane, where it becomes active upon GTP binding [8]. Each Rab contains a hypervariable region, which is involved in membrane tethering and is specific to both the Rab and the target membrane where they carry out their function [9,10]. Once associated with the target membrane, the Rab acts as a scaffold protein and interacts with a variety of downstream effectors to complete its function. Following this, hydrolysis of GTP to GDP is driven by GTPase activating proteins (GAPs) to inactivate the small GTPase. GDP dissociation inhibitors (GDIs) then retrieve the GDP-bound Rab from the membrane to solubilise it in the cytosol so that the cycle can begin again. Rabs also undergo the important post-translational modification of prenylation, which controls its membrane association [11]. Prenylation is mediated by the Rab escort protein (REP), which binds to the Rab and presents it to the Rab geranylgeranyltransferase (GGTase) for double prenylation [8].

## 2. Rab GTPases and Neurodegeneration

Rabs have been linked to multiple different diseases including cancer, diabetes, and various genetic disorders such as Griscelli syndrome [13]. Rabs have also been implicated in several neurodegenerative diseases, many of which have aberrant protein folding, vesicle trafficking, and secretion as key molecular pathologies [14,15]. These GTPases could also be involved in the lifecycle of viruses such as the Herpes Simplex Virus (HSV), where there is increasing evidence suggesting that HSV re-activation in the brain could lead to cognitive impairment and the development of AD [16]. Indeed, increasing Rab levels have been found to ameliorate phenotypes in a variety of disease models. For example, overexpression of the yeast Rab1 homolog Ypt1p rescues dopaminergic neuron loss caused by α-synuclein in animal models of Parkinson’s disease (PD) [17], whilst overexpression of Rab1A in rodent models of PD was found to prevent pathological Golgi fragmentation and rescue motor deficits [18]. Similarly, Rab11 overexpression significantly rescues PD- and Huntington’s-disease-related phenotypes in fly models, including neurodegeneration and behavioural deficits [19,20]. Given the localization and physiological function of different Rab GTPases, they are thought to play different roles in disease pathogenesis, thereby having therapeutic relevance (Table 1). This review focuses on the therapeutic targeting of Rab GTPases in AD, owing to promising results from Rab modulation.

AD is the most common form of dementia—currently accounting for ~60–70% of cases [116]. Clinical symptoms of AD include gradual memory loss, cognitive decline, and mood changes. AD is characterised by two main neuropathological hallmarks: amyloid-beta (Aβ) plaques, and hyperphosphorylated tau neurofibrillary tangles (NFTs). Currently available treatments can slow the progression, but do not alter the course of the disease. However, the complex pathways leading to Aβ plaque and NFT formation rely heavily on neuronal protein trafficking, processing, and exocytosis. Indeed, several Rab GTPases have been implicated in these pathologies and are thought to play important roles in the aberrant processing pathways that lead to the aggregation and accumulation of these toxic proteins [117].

## 3. Rab GTPases and the APP Processing Pathway

### 3.1. APP Processing Pathway

**Figure 3:**
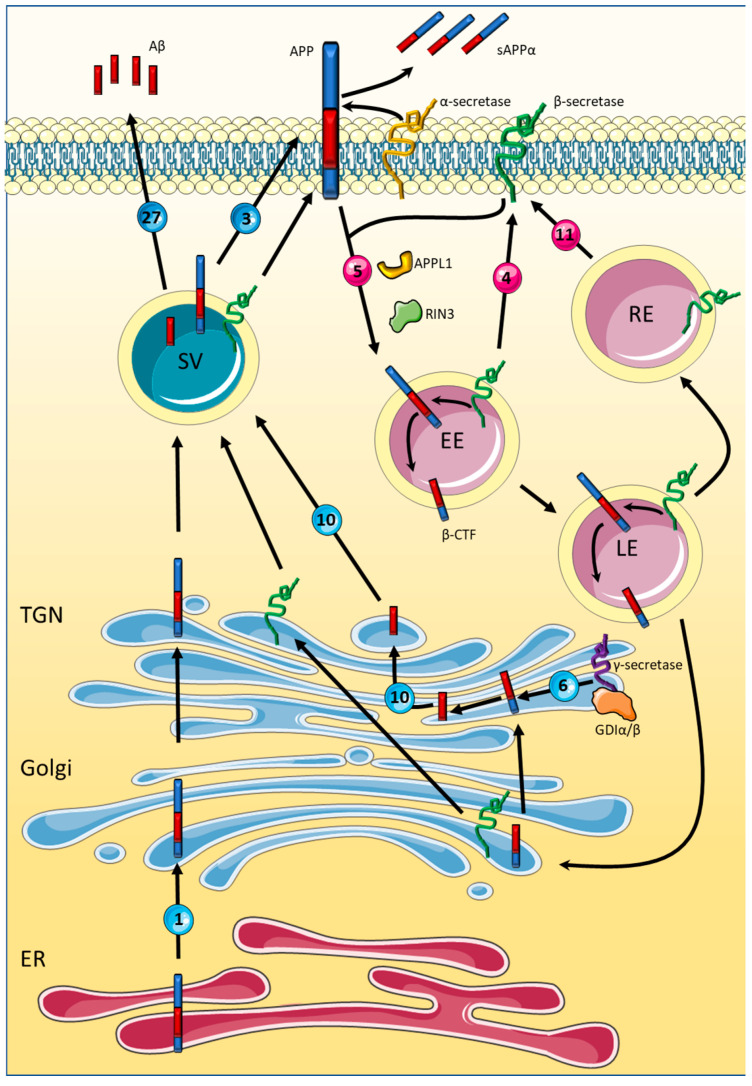
Rab-mediated amyloid precursor protein metabolism. De novo amyloid precursor protein (APP) is produced with the endoplasmic reticulum (ER) and transported to Golgi for protein maturation via a Rab1 trafficking pathway. Upon passage through the Golgi and trans-Golgi network (TGN), APP resides with the plasma membranes. APP may be processed via the non-amyloidogenic route via α-secretase producing soluble APPα which is released into the extracellular environment. Alternatively, APP along with β-secretases may be internalised via Rab5-dependent endocytosis. Once in the acidic internal compartments of early and late endosomes (EE/LE) the cleavage of APP via β-secretases generates the β-C-terminal fragment (β-CTF). β-secretases is in turn recycled to the plasma membrane either directly via Rab4-mediated traffic, via recycling endosomes (RE) dependent on Rab11 trafficking or trafficking alongside the β-CTF to the Golgi. Within the Golgi and TGN, the γ-secretase complex facilitated by its association with Rab6 processes the β-CTF into β-amyloid (Aβ) which is trafficking into secretary vesicles via Rab10 and released via Rab27/Rab3-dependent process, alongside post Golgi trafficking APP and β-secretase. Also shown is the faciliatory role of Ras and Rab interactor 3 (RIN3) and adaptor protein, phosphotyrosine interacting with PH domain and leucine zipper 1 (APPL1) on Rab5-mediated endocytosis as well as the association of GDP dissociation inhibitors (GDIα/β) with the γ-secretase component presenilin-1. The alternative processing of β-CTF into Aβ within the lysosomes is not shown in this schematic. Rabs more strongly associated with secretory pathways are shaded in blue while those more strongly associated with endosomal pathways are shown in red.

Aβ plaque formation is thought to occur through the progressive aggregation of Aβ peptides, thereby forming oligomers, fibrils, and finally Aβ plaques. Aβ peptides are produced through the sequential cleavage of the amyloid precursor protein (APP). APP has several physiological functions including neuronal survival, synaptogenesis, synaptic plasticity, and neuronal excitability [118], and can be cleaved by two different pathways. The first is considered to be non-amyloidogenic and takes place at the cell surface membrane by an α-secretase. The amyloidogenic pathway requires sequential cleavage by β- and γ-secretases and leads to the production of toxic Aβ peptides. The primary β-secretase responsible for APP cleavage is thought to be Beta-secretase 1 (BACE1) [119], which internalises within endosomes, where it interacts with APP [120]. This accumulation of BACE1 is regulated by the subsequent removal of BACE1 via retrograde endosomal to Golgi transport [121], as well as recycling from the endosomes to the plasma membrane [38]. Following BACE1 cleavage, a γ-secretase protein complex cleaves the APP-produced β-C-terminal fragment (β-CTF) to produce the toxic Aβ peptides [122], which, although largely debated, likely occurs in either the Golgi apparatus [123] or within lysosomes [124]. As the endocytic pathway is essential for the APP amyloidogenic processing pathway, it is therefore unsurprising that several Rab GTPases known to localise and function within the endocytic pathway have been implicated in AD (See Figure 3). Below we discuss several Rabs involved in endocytosis that have been linked to AD, as well as those directly linked to APP processing.

### 3.2. Rab5

Rab5 is one of the better characterised Rab GTPases and is central to the early endocytic pathway [47,48,49], localising primarily to early endosomes [27]. It is upregulated in several different vulnerable brain areas in AD; the basal forebrain, frontal cortex, and hippocampus in the early stages of AD, and even in mild cognitive impairment (MCI) [44,45]. One of the earliest alterations in AD pathology is the enlargement of Rab5+ early endosomes [50], which can be induced by both APP and β-CTF through the activation of Rab5 [125]. The Rab5 effector APPL1 (adaptor protein, phosphotyrosine interacting with PH domain and leucine zipper 1) is a key protein in β-CTF-dependent endosomal dysfunction and is thought to be recruited by β-CTF to Rab5-positive endosomes where it stabilises the GTP-bound active form of Rab5 associated with the endosome membrane [126]. Through this mechanism, Rab5 becomes overactivated causing pathological endosomal dysfunction and enlargement [126]. Rab5 also upregulates the APP processing pathway when overexpressed in murine cells, significantly increasing the production of both Aβ40 and Aβ42 [52].

These interactions between Rab5 and the APP processing pathway provide a significant therapeutic opportunity and studies have shown benefits from Rab5 modulation. Amyloid precursor protein binding protein 1 (APP-BP1) controls the S to M transition during the cell cycle, and has been shown to bind to APP, inducing neuronal apoptosis [127]. In primary neuronal cultures, exogenous expression of APP-BP1 or APP increases protein levels of Rab5, increasing the number of endosomes and causing endosomal enlargement. Rab5 has been shown to co-localise with and bind to APP-BP1, indicating a possible role in neuronal apoptosis, and subsequent expression of a dominant negative Rab5 further validated this by rescuing apoptosis [128]. Furthermore, a dominant negative Rab5 mutant was able to rescue disrupted axonal transport caused by APP and β-CTF in *Drosophila* [125]. Taken together, these studies provide evidence that the modulation of Rab5 activity could rescue some of the AD defects caused by endosomal dysfunction.

Additionally, Rab5 effectors also play a role in APP processing and could be targeted. Ras and Rab interactor 3 (RIN3) is a GEF that functions as an effector for Rab5. RIN3 has been identified as a risk factor for both early-onset AD (EOAD) and late-onset AD (LOAD) [129,130]. Using the APP/PS1 mouse model of AD, it was found that RIN3 mRNA and protein levels are increased in the hippocampus and cortex from only 3 months of age, which greatly precedes the formation of Aβ plaques in this model—which usually occurs at ~6–7 months [131]. Overexpression of APP in cultured rat basal forebrain cholinergic neurons (BFCNs) leads to enlarged endosomes and selective upregulation of RIN3 [125], which may indicate a role for RIN3 in this pathology given its links to Rab5 [131]. Notably, RIN3 interacts with the AD risk factor proteins bridging integrator 1 (BIN1) [132] and CD2-associated protein (CD2AP) [133,134], recruiting them to early endosomes. RIN3 and CD2AP function together to increase toxic APP β-CTF production by disrupting BACE1 and APP trafficking [131].

### 3.3. Other Endosomal Rabs

Additional endosomal Rab GTPases have been implicated in AD. Rab4 is another early endosome Rab upregulated in AD, although not MCI. However, Rab27, which localises to secretory vesicles and the plasma membrane, is upregulated in MCI [45]. Meanwhile, increased levels of Rab6 have been identified in the hippocampus, entorhinal cortex, and temporal cortex during early-stage AD [57], correlating with an increase in binding immunoglobulin protein (BiP), a key marker of ER stress and the unfolded protein response (UPR). Furthermore, Golgi localised Rab6 [55] could play a role in ER stress, increasing retrograde protein transport to the ER, overloading the ER and causing the accumulation of proteins [57]. Indeed, expression of a dominant negative Rab6 mutant increased secretion of the soluble ectodomain s-APPα, which is formed by cleavage of APP by α-secretase, indicating a role for Rab6 in the non-amyloidogenic pathway. It is hypothesised that the dominant negative Rab6 mutant could be facilitating anterograde transport or inhibiting retrograde transport to allow APP to move into the non-amyloidogenic pathway [59]. Rab6 also regulates the membrane association of presenilin 1 [58], mutations of which are common causes of familial AD, suggesting a further pathogenic role for Rab6 in AD.

Rab10 has recently been linked to AD pathology due to its upregulation in the temporal cortex of patient brains [76]. Furthermore, overexpression of Rab10 in mouse neuroblastoma cells significantly increases Aβ production with full length APP levels being unaffected, suggesting that Rab10 function occurs in the later stages of the amyloidogenic pathway [76]. Indeed, levels of sAPP were unaffected in these cells, indicating that Rab10 likely acts after β-secretase cleavage, and instead during γ-secretase cleavage [76]. Importantly, knockdown of Rab10—as well as Rab4 and Rab6A—significantly reduced Aβ levels [38], providing evidence for the therapeutic potential of Rabs in the later stages of Aβ production.

An RNAi screen identified several Rabs as regulators of Aβ and sAPPβ synthesis [38]. When silenced, Rab11A significantly decreased the production of the two proteins. Further experiments identified a crucial role for Rab11 in the recycling of BACE1 through Rab11-positive recycling endosomes [38]. Rab11 also regulates the axonal transport and sorting of BACE1 to presynaptic terminals [135], and contributes to the impaired transcytosis of APP to the soma, a pathology found in familial AD mutant neurons [136]. Critically, in a proteomic study of AD cases, Rab11 expression was notably increased compared to controls [137], consistent with a facilitatory upregulation of the enzyme as part of an overactivation of the amyloidogenic pathways. As Rab11 has shown great potential for other neurodegenerative diseases, these studies suggest that the modulation of Rab11 within the context of AD could have similar benefits.

### 3.4. ER, Golgi, and Exocytic Rabs

Also involved in the early-stage trafficking of APP is Rab1B, which contributes to protein trafficking between the Golgi and ER [21]. Expression of a GTP-binding defective Rab1B mutant in transformed human embryonic kidney cells inhibited the maturation of APP, thought to occur in the Golgi, providing evidence for the role of Rab1B in the transport of APP from the ER to the Golgi for post-translational modifications [26]. Furthermore, the dominant negative mutant, Rab1B^N121I^, reduced the secretion of sAPPα from the non-amyloidogenic pathway, and sAPPβ produced by the amyloidogenic pathway, providing further evidence that both pathways are reliant on ER to Golgi transport at an early stage. However, the production of Aβ was also inhibited [26], suggesting another possible therapeutic target (see below for consequences on tau homeostasis). Rab39B is enriched in neuronal cells and localises to the ER, Golgi, and recycling endosomes. Rab39B was shown to co-localise with Aβ plaques in AD cases and Lewy bodies, in dementia with Lewy bodies (DLB) cases, indicating sequestration within these protein aggregates [138]. Whilst there was no change in the cytoplasmic Rab39B levels in AD cases, there was a reduction in DLB cases. This could be due to an increase in active, membrane-associated Rab39B as a compensatory mechanism, or to the sequestration of Rab39B to protein aggregates [138].

Rab3A is also downregulated in AD brains [39], and further work found that Rab3A knockdown decreased the production of Aβ and sAPPβ [38]. Rab3A is involved in exocytosis and is crucial for the formation of vesicles involved in anterograde fast axonal transport of APP to the subsequent cleavage pathways [40]. A study of AD patients identified increased levels of Rab3, and also Rab7, in the CSF, which could indicate a use for them as biomarkers for AD diagnostics, further demonstrating how varied the therapeutic opportunities are for Rabs [41].

In addition to the reported interactions of presenilin 1 with Rab6 [58] and Rab11 [79], the γ-secretase component also directly binds GDIα/β in the ER [58,139]. This association has been speculated to direct GDIs to membranes prior to the recovery of Rab–GDP complexes and thus may serve to prime GDIs for interaction with inactive Rab proteins and subsequently enter them into a reactivation cycle, a process which is anticipated to be enhanced by familial AD presenilin mutations [139]. Furthermore, several proteomic studies have reported the significant upregulation of GDIα and GDIβ in AD brains compared to controls [137,140]. Given that GDIα/β are the predominate regulators for the extraction of Rab–GDP complexes from the membrane prior to reactivation via GEF-mediated GTP exchange [141], such upregulation likely enhances the rate of membrane re-engagement, in turn facilitating the overactivation of membrane trafficking in favour of the amyloidogenic metabolism of APP. Collectively the data highlights GDIs as prominent targets of therapeutic potential.

## 4. Rab GTPases and Tau Pathology

Intracellular NFTs are another hallmark of AD pathology, caused by the progressive accumulation and aggregation of hyperphosphorylated tau. The occurrence of tau aggregates in AD places this disorder in a family known as tauopathies, which also includes frontotemporal lobar degeneration with tau pathology (FTLD-tau). Although not considered a primary tauopathy, PD also presents with tau pathology. The tau protein is encoded by the *MAPT* gene, which is located on the long arm of human chromosome 17. Once translated, the mRNA can undergo splicing to form six different isoforms, followed by several different post-translational modifications, including phosphorylation, acetylation, glycosylation, and nitration [142]. Under physiological conditions, approximately 80% of tau in the cell is bound to the microtubules of axons, providing structural integrity and stability [143,144]. The tau protein also plays an important role in regulating axonal transport and cargo shuttling [143]. Pathological hyperphosphorylated tau, as found in tauopathies, dissociates and destabilises microtubules, causing synaptic impairment and subsequent neurodegeneration. Hyperphosphorylated tau can aggregate to form paired helical filaments, which continue to associate in AD to form NFTs. Arguably, tau pathology correlates better with AD-associated disease progression and cognitive decline than Aβ plaques [145,146,147]. Thus, it is possible that targeting tau pathology may provide better clinical outcomes than those obtained with strategies targeting Aβ plaques. Notably, several Rab GTPases have already been implicated in tau pathology.

As tau pathology is present in multiple neurodegenerative diseases, it is not surprising that other proteins linked to tau pathology are common among these disorders. Leucine-rich repeat kinase 2 (LRRK2) mutations are a common genetic cause of PD [148], whilst the R1628P LRRK2 variant increases the risk of AD [149]. Several Rab GTPases, including Rab10, are phosphorylated by LRRK2, which is a member of the leucine-rich repeat kinase family [150]. Furthermore, phosphorylated Rab10 (potentially via LRRK2) has been identified within NFTs, partially co-localising with phosphorylated tau. This suggests that phosphorylated Rab10 may play a role in the pathology and accumulation of NFTs [78], although it is still undetermined if modulation of this Rab could reverse disease phenotypes.

Although NFTs are an intracellular hallmark of AD, pathological tau is also secreted into the CSF. Whilst this secretion could be viewed as somewhat beneficial, as it is clearing misfolded, toxic proteins from the cell, preventing aggregation, it actually correlates closely with neuronal cell death and cognitive decline in tauopathies [151]. Furthermore, secreted tau has been suggested to propagate through the brain once secreted via a prion-like mechanism, although this is a relatively new concept and somewhat controversial, with more studies required [152]. Regardless, secretion of tau to the CSF has been confirmed and several Rabs have been implicated in this process, which could potentially be targeted.

Golgi fragmentation occurs as a result of AD and contributes to defects in neuronal trafficking [153]. Notably, these alterations correlate well with the accumulation of hyperphosphorylated tau into NFTs. Rab1A is involved in ER to Golgi trafficking [154], and its knockdown significantly increases Golgi fragmentation in primary cortical neurons and HeLa cells, leading to an increase in tau secretion [155]. Tau secretion may also be regulated by Rab7A, which is upregulated in AD and mild cognitive impairment (MCI) [44,45]. Rab7A is localised to late endosomes [27] and lysosomes [60], playing key roles in the progression of endocytosis [49] and lysosomal biogenesis [60]. Rab7A is also involved in the maturation of autophagosomes, essential for autophagy [63]. However, the suspected role of Rab7A in AD is central to tau pathology. Rodriguez et al. demonstrated that overexpression of a dominant negative Rab7A mutant significantly decreased the level of tau secreted from both HeLa cells and primary cortical neurons [65]. This was further validated by overexpression of a constituently active form of Rab7A, which caused an increase in tau secretion [65]. These results demonstrate that Rab7A is crucial to the regulation of tau secretory pathway. The upregulation of Rab7A observed in AD brains [51] could indicate a potential mechanism by which tau secretion is increased, correlating with cell death, toxicity, and further propagation through the brain. Thus, modulation of Rab7A activity could be an interesting and novel therapeutic approach for AD.

Tau accumulation can be triggered by high levels of glucocorticoids, the main stress hormones, resulting in neuronal atrophy, cognitive impairment, and synaptic dysfunction [156,157,158]. This accumulation can occur due to the breakdown and dysfunction of the degradative pathways, preventing the degradation of misfolded, pathological tau and clearance from the cell. Rab35 is involved in endocytic recycling of receptors and other membrane proteins, localising to the plasma membrane [109]. It is a key protein in the endosomal sorting complex required for transport (ESCRT) pathway, which is involved in the degradation of ubiquitylated proteins in multivesicular bodies (MVBs). Tau has been detected in these MVBs and shown to be degraded via lysosomes mediated by the ESCRT pathway. Vaz-Silva and colleagues demonstrated that glucocorticoids suppress Rab35 expression, which in turn inhibits the degradation of tau via the ESCRT pathway, leading to accumulation and toxicity. Importantly, overexpression of Rab35 upregulates the degradation of tau and rescues the associated neuronal atrophy caused by the toxic accumulation of tau [111].

Rab effectors have also been linked to tau pathology. BIN1 is a trafficking protein that has been identified as an AD risk factor [159], in particular, interacting with the processing pathways of tau aggregates [132]. RIN3, a Rab5 guanine exchange factor (GEF), has been shown to recruit BIN1 to the early endosomes, as previously discussed, where it is thought they work together to upregulate levels of hyperphosphorylated tau [131]. However, the exact mechanisms of how these Rab effectors and other trafficking proteins interact with tau pathology still requires further research, although they may provide an interesting therapeutic target that could be used to modulate tau pathology.

Perhaps the most prominent GEF associated with neurodegeneration is Chromosome 9 open reading frame 72 (C9orf72). Familial hexanucleotide GGGGCC repeat expansion in the C9orf72 gene accounts for a third of familial amyotrophic lateral sclerosis and a quarter of frontotemporal dementia (FTD) cases, leading to its haploinsufficiency and inducing the formation of RNA-rich nuclear aggregates [160]. Whilst the association of C9orf72 hexanucleotide expansions with AD is controversial [161,162,163,164], meta-analysis supports an increased risk for AD [165]. In any case, C9orf72 expansion carriers present with robust AD-like tau pathology [166] and in AD, independent of gene mutation, the C9orf72 protein accumulates within hyperphosphorylated tau bearing dystrophic neurites associated with Aβ plaques [167]. Together the studies suggest dysfunction of C9orf72 as being common to AD and likely contributory to the mechanisms leading to the accumulation of tau pathology. Critically, C9orf72 associates with a number of Rab proteins [168] and is an effector of Rab1A [169]. C9orf72 also complexes with SMCR8 and WDR41, for which both Rab8A and Rab39 GEF [69], and Rab8A and Rab11B GAP activity [170] has been reported. Functionally, the modulation of Rab activity via C9orf72 appears to regulate autophagic flux [69,169,171], but also the surface expression of glutamate receptors [172], thus implicating a dysfunction of C9orf72 as contributory to both pathological tau accumulation and disease-mediated changes in neuronal signalling.

## 5. Pharmacological Targeting of Rab GTPases

As with all central nervous system diseases, the development of effective interventions is challenging. This is, in part, due to the low bioavailability of pharmacological drugs in the brain caused by the impermeably of the blood–brain barrier, but also due to complex issues relating to the targeting of specific brain regions, cell types or even neuronal subtypes. Furthermore, drug development towards the modulation of Rab proteins can be considered additionally problematic due to the largely as yet unresolved nature of their endogenous regulation, in addition to the high degree of homology amongst Rab family members and with other small GTPase families such as Rho and RAS proteins. Yet the integral involvement of Rab proteins in the pathology of, not only AD, but in numerous other diseases, including other neurodegenerative disorders, makes them an enticing target for therapeutics. At a conceptual level, there exist multiple routes in which the activity of Rab proteins can be modulated, including the targeting of (1) Rab protein binding of GTP and subsequent activation, (2) processes underlying Rab prenylation and other post-translational modifications, and (3) Rab effector proteins. Below we highlight several approaches which have yielded relative success and may, in turn, be built upon for future therapeutic development.

### 5.1. Competitive Antagonists and Allosteric Modulators of Rab Proteins

Of all the mechanisms employed to pharmacologically target enzymatic activity, competitive antagonism of nucleotide engagement is by far the most common. However, small GTPs such as Rhos, RACs, and Rabs have an extremely high affinity for GTP, which has largely ruled out the use of such an approach for Rab-targeted drug design. One exception to this is CID1067700, a small molecule identified from >3000 compounds via high-throughput screening, as the sole candidate capable of competing GTP for Rab binding [173]. This action, although greatest for Rab7, was largely non-specific, with CID1067700 inhibiting other Rabs and the related GTPases Rho and RAC. In addition, the induced antagonism was highly sensitive to GTP concentrations in vitro, raising concerns of efficiency at physiological GTP concentrations. Nevertheless, several studies have reported favourable outcomes of Rab7 inhibition following CID1067700 intervention in rodent models of ischemic stroke [174] and lupus [175]. Given the increased Rab7a expression in AD and its role in the secretion and potential spread of tau pathology, an evaluation of CID1067700 administration in pre-clinical models would seem warranted, with the caveat that inhibition of autophagic flux may negatively impact upon Aβ and, indeed, the viability of pathology-bearing cells. Regardless of the therapeutic relevance of such a compound, these studies nevertheless set a precedent for the effective targeting of specific Rab proteins via competitive GTP antagonism.

Akin to the limited success of direct Rab antagonism, few allosteric modulators of Rab proteins have been reported. Nonetheless, recent structural analyses of the small GTPases RAS, Rab1, and Rab11 have facilitated the identification of allosteric binding sites with the potential for modulating GTP/GDP binding. Whilst structurally conserved, these sites are not identical in sequence and may provide scope for the development of specific ligands for the differential targeting of many of the Rab protein family members [176,177]. Indeed, several small molecules capable of interacting with such sites in Rab11 have been reported, with the drugs displaying preferential binding when Rab11 is in various states of nucleotide association, presumably enabling the potential for allosteric activation or inhibition [177]. Given the multiple roles for Rab11-mediated trafficking in Aβ production, and its upregulated expression in AD (as above), determining the consequence of these drugs on APP metabolism in cellular systems may uncover potential therapeutic benefits.

Different studies report on the potential of small molecules to act as allosteric activators of Rabs, as these compounds act to stabilise the binding of GTP to Rabs and, therefore, can be considered to facilitate prolonged Rab activation. Given that a number of studies appear to a support a protective role for the overexpression of specific Rab proteins in various neurodegenerative diseases, such compounds may hold promise in stimulating Rab proteins reportedly inhibited as a consequence of the AD process. However, to date, work in this line of investigation has been limited and has primarily focused on a small library of compounds, initially identified from a screening of 20,000 molecules [178]. From three chemical families (nicotinic, indole, and salicylic acids) 43 compounds with activation properties across the GTPase families (Rac Rho, Rabs) have been identified, with the salicylic acid compounds CID 3338522, CID 7721337 and CID 1508555, being relatively selective for Rab2 and or Rab7 activation within the nM range [179]. Despite scant research in this area, advances in understanding of the conformational changes in Rab proteins following changes in activation states, and the determination of potential allosteric sites for the stabilisation of such conformations, are likely to bring new renewed potential for the development of selective allosteric activators and inhibitors in the future [180].

### 5.2. Prenylation Inhibitors

Given that many Rab proteins are reported as dysfunctional and largely overactive in AD, the uses of selective modulators targeting one specific Rab family member may not meaningfully impact their facilitatory role in AD pathogenesis. Therefore, a more broad-spectrum approach may arguably be more beneficial in reducing this pathological overactivation. The generalised downregulation of Rab activity may be achieved by the selective targeting of common modifiers to all Rab proteins. Indeed, there is evidence to justify the targeting of upstream proteins, as elevated levels of the isoprenoids geranylgeranyl diphosphate (GGDP) and farnesylpyrophosphate (FPP)—essential substrates of FTase, GGTaseI, and Rab GGTase II prenylation—have been found in AD brains [181]. In cancers, where the overactivation of small GTPase proteins is also observed, efforts have largely focused on the inhibition of prenylation. However, many of the lead compounds have been developed to inhibit prenylation enzymes such as farnesyl transferase and/or GGTase I and not Rab GGTase II, prioritising the inhibition of Rac and Rho small GTPases, as opposed to Rabs [182,183,184]. Nevertheless, [3-PEHPC 2-(3-pyridinyl)-1-hydroxyethylidene-1,1-phosphonocarboxylic acid] and [3-IPEHPC 2-Hydroxy-3-imidazo [1,2-a]pyridin-3-yl-2-phosphonopropionic acid], as well as psoromic acid, have been identified as specific Rab GGTase inhibitors and have been used in a variety of assays to establish effective inhibitory effects on Rab activity [185,186,187,188]. Despite their proven cellular efficacy, the inhibition of Rab activity via such compounds in AD models remains unexplored. As an alternative to the direct inhibition of Rab GGTases, the upstream inhibition of enzymes responsible for the production of GGDP also offers the potential for broad-spectrum Rab inhibition. Interestingly the depletion of isoprenoid GGDP, and thus Rab inhibition, has been associated with a pleiotropic effect of statins which can reduce Aβ production in cell lines, independent of any effects on cholesterol [189]. Despite evidence for the effectiveness of brain isoprenoid GGDP depletion and small GTPase inhibition in vivo at clinically relevant statin concentrations [189], and early reports of the protective effect of statins against AD [190,191,192], more recent studies have had contradictory outcomes [193]. Notably, specific geranylgeranyl diphosphate synthase inhibitors have recently been developed [194,195]. When tested in AD models, these may prove more readily beneficial in contrast to specific statins which have a range of pharmacological actions and, consequently, may not act via a direct consequence of their efficacy towards Rab inhibition [196].

### 5.3. Modulators of Rab Effectors

Further potential for Rab protein modulation may lie in the targeting of other Rab effectors such as GEFs, GAPs, and GDIs. Yet, despite the potential for their targeting, the identification of these effectors is generally lacking, particularly for GEFs, for which there exists a significant proportion of Rab family members without a characterised GEF. In recent years, however, progress has been made in identifying proteins with GEF activity, with DENN-domain-containing proteins, by far, forming the major group of GEF proteins within a diverse list of activators [197,198]. In contrast, the prominence of Tre-2/Bub2/Cdc16 (TBC) domains in the majority of GAPs has allowed for a large number of TBC-containing proteins to be screened for GAP activity against many different Rab proteins, enabling a rather comprehensive list of molecules capable of deactivating Rab proteins via the facilitation of GTP hydrolysis [199]. Unfortunately, at present, little is known about the potential modulators of Rab GEF and GAPs. Nevertheless, considerable work has been conducted into RAS and Rho GAP/GEF inhibitors, which have identified a variety of mechanistic approaches for influencing regulatory processes [200] that could, in turn, be translated for Rab modulation in the future.

Instead, targeting upstream regulatory kinases of Rab proteins and their effectors may be more immediately accessible. To date, direct Rab protein phosphorylation has been attributed to cyclin-dependent kinase 1, leucine-enriched kinase 1 and 2, multiple protein kinase C isoforms, TANK-binding kinase 1, transforming growth factor-β-activated kinase-1, and tyrosine kinase Src [198]. Rab phosphorylation typically interferes with their association with particular effectors and, consequently, can facilitate or inhibit Rab-mediated trafficking, depending on the nature of the disrupted interaction. For example, phosphomimetic studies of LRRK2 substrate Rabs demonstrate decreased associations with GDI, which in turn can be anticipated to slow the cycling of membrane-bound Rab–GDP complexes into the cytoplasm for reactivation [201]. Amongst those regulated by LRRK2 are Rab3 and Rab10, which have been highlighted as overactive and facilitatory of Aβ exocytosis and, as such, facilitating LRRK2 phosphorylation may be considered a potential point for pharmacological intervention. However, given the association of LRRK2 mutations with familial PD [202], and its kinases reported overactivity in idiopathic cases [203], establishing a safe therapeutic window for its modulation may not be possible. Alternatively, emerging regulatory pathways for GAP proteins may offer improved potential for exploitation, as highlighted by the recent characterisation of lemur tyrosine kinase 1 (LMTK1). Both Rab11-GAP TBC1D9A and Rab1-GAP TBC1D20 are regulated via LMTK1 interactions with direct phosphorylation of these GAPs assumed responsible for their enhanced functionality and, consequently, the deactivation of Rab proteins [204]. Interestingly, a single nucleotide polymorphism associated with the reduced expression of the LMTK1 gene (AATK1) has been identified as an FTD risk factor within an Italian cohort [205], suggesting that abnormal GAP regulation may be a contributory factor in tau pathology, as well as potentially in the amyloidogenic processing of APP.

Likewise, the upstream regulation of GEFs may be exploitable, as G-protein-coupled-receptor- (GPCR) mediated regulation of Rab39B within striatal neurons has been demonstrated via GPR-52 in a cyclic-AMP-dependent manner [206]. Thus, targeting of GPCRs, and potentially modulation of cAMP levels, may be able to modulate GEF activity, with both inhibitory and excitatory outcomes for selective Rab proteins. Such work suggests the manipulation of upstream receptors, kinase, and/or second messengers as points of potential therapeutic intervention, with the hope that, in the future, further delineation of these regulatory pathways—and potential others related to additional Rab proteins—may expand the scope of approach.

### 5.4. Pathogen-Secreted Rab Effectors

A final consideration towards identifying approaches for the modulation of Rab activity can be taken from infectious pathogens. In order to maintain their intracellular presence and to avoid degradation, invading bacteria and viruses secrete an array of molecules targeting different molecular components of endosomal trafficking, effectively hijacking these routes to meet their own requirements [207]. Many of these secreted molecules directly interact with Rab proteins and their effectors. For example, *Chlamydia trachomatis* produce an inclusion membrane protein (CT2999) which binds and sequesters a number of active Rab–GTP complexes, consequently suppressing host cell endosomal trafficking [208]. Likewise, *Coxiella burnetiid* secrete both a prokaryotic kinase (Coxiella Ser/Thr kinase) which interacts with the Rab7 GAP TBC1D5 to enhance Rab7 activity, which is required for the production of Coxiella-containing vacuoles [209] and also Coxiella vacuolar protein F, which directly associates with Rab26 and consequently enhances host cell autophagic degradation [210]. In fact, various invading pathogens have evolved an arsenal of molecules capable of subverting host Rab-mediated trafficking. For example, *Legionella pneumophila* secretes molecular mimics of Rab1 GEF and GAPs thus allowing for the modulation of the Rab1 activation/deactivation cycle [211]. Even multiple Rab-interacting non-structural proteins secreted by severe acute respiratory syndrome coronavirus 2 (SARS-CoV-2) have been highlighted as prospects for drug repurposing [212]. Although as yet unexploited, the array of pathogen-generated molecules capable of selectively manipulating intracellular trafficking routes may yield novel and effective means of addressing both the overactivation and suppression of Rab-mediated trafficking as part of the pathology of AD and other diseases.

Collectively, the studies reviewed above suggest that significant progress has been made in targeting the once-thought ‘undruggable’ Rab protein network. In the future, advances in the characterisation of endogenous Rab regulation, the identification of additional Rab interaction partners, and further study of the role of Rabs in infectious diseases may provide a critical insight into targeting Rabs. Ultimately, such work may aid in generating a tool box of compounds with improved specificity for single families of GTPases (Rabs Cf. Rho Cf. RAS GTPases) and the capability of either selectively targeting individual Rab proteins or particular functional trafficking routes (endosomal Cf. exosomal, etc.), which may one day progress to the clinic.

## 6. Final Considerations

The studies discussed herein provide strong evidence that Rab GTPases are not only heavily involved in AD pathology, but also that modulation of Rab expression and activity could rescue associated defects and toxicity. Focusing on such a large protein family provides many different therapeutic targets that could be exploited at many stages of the disease. It also offers the potential for a multifaceted approach to treatment, incorporating different Rabs to target multiple aspects of AD pathology at once. Although therapeutic targeting and modulation of these small proteins has proven to be difficult in the past, the approaches outlined above, aimed at targeting Rab GTP binding, post-translational modifications, and Rab effectors, have had promising early success and could be developed further into effective therapeutic strategies. Before these strategies can reach the clinic, the common issues with targeting neurodegenerative diseases will need to be addressed, such as the crossing of the blood–brain barrier, as well as more Rab-specific concerns including a lack of specificity and low binding affinities. Whilst the approaches summarised in this review have had success, there is much more research required in pre-clinical models to fully understand potential off-target effects and how these modulations could affect other aspects of AD pathology as a whole. It is vital that research in this field continues to dig deeper into the various pathological roles of Rabs and their effectors in AD, and in the wider context of neurodegeneration, to further elucidate the complex mechanisms involved and discover more potential therapeutic targets.

## Figures and Tables

**Table 1:** Rab GTPases and their known localisations and functions under physiological conditions are summarised here, including their various links with Parkinson’s Disease (PD), Huntington’s Disease (HD), Dementia with Lewy Bodies (DLB), and Alzheimer’s Disease (AD). Endoplasmic reticulum (ER), trans-Golgi network (TGN), endoplasmic reticulum Golgi intermediate compartment (ERGIC), amyloid beta (Aß), amyloid precursor protein (APP), cerebral spinal fluid (CSF), knockdown (KD), huntingtin (Htt), leucine-rich repeat kinase 2 (LRRK), neurofibrillary tangles (NFT), Htt-associated protein 40 (HAP40).

Rab GTPase	Localisation	Function	Link with Neurodegenerative Diseases	Citations
**Rab1A/B**	ER Golgi	Biosynthesis Protein transport Autophagy Cell signalling Migration and proliferation	PD Impaired by α-synuclein Overexpression rescues defects AD Involved in Aß production	[17,18,21,22,23,24,25,26]
**Rab2A**	ER ERGIC Golgi Autolysosome	Protein anterograde and retrograde transport Autophagy and lysosomal degradation	HD Huntingtin suppression increases Rab2+ vesicle transport	[27,28,29,30,31]
**Rab3A/B**	Secretory vesicles	Exocytosis	PD α-synuclein interaction Overexpression rescues and protects against dopaminergic neuron loss DLB Rab3 levels decreased in Lewy body dementia—correlates with cognitive decline HD Overexpression rescues impaired vesicle docking and reduces reactive astrocytes AD Regulates Aß production Downregulated in AD brains Involved in APP transport Increased in AD CSF	[24,32,33,34,35,36,37,38,39,40,41]
**Rab4A**	Early endosome	Protein sorting to both recycling and degradative pathways	AD Upregulated HD Involved in Htt axonal trafficking—disrupted in HD Overexpression rescues synaptic defects, lifespan, and locomotor defects	[42,43,44,45,46]
**Rab5**	Early endosome Plasma membrane	Early endosome regulation	AD Upregulated and causes enlarged endosomes—early AD pathology Increases Aß production HD Interacts with Htt via HAP40 Modifies mutant Htt toxicity and aggregation	[27,44,45,47,48,49,50,51,52,53,54]
**Rab6A**	Golgi	Targeting of secretory vesicles during exocytosis	AD Upregulated Interaction with presenilin 1 Dominant negative mutant decreases Aß levels	[55,56,57,58,59]
**Rab7**	Late endosome Lysosome Phagosomes	Late endosome regulation Lysosome biogenesis Phagosome maturation Autophagy	PD Increases autophagy, reduces cell death, and rescues locomotor deficits HD Reduced Htt impaired Rab7+ vesicle motility AD Upregulated Overexpression decreases tau secretion	[27,31,44,45,49,51,60,61,62,63,64,65]
**Rab8**	Plasma membrane Synaptic vesicles Autophagosomes	Polarised membrane trafficking Cell morphogenesis Autophagy	PD Overexpression rescues dopaminergic neuron loss Increases α-synuclein aggregation—reduces toxicity Mediates transport of α-synuclein extracellular vesicles HD Rescues HD defects and reduces neurodegeneration Promotes HTT aggregation	[24,66,67,68,69,70,71,72]
**Rab10**	ER Endosomes Phagosomes GLUT4 vesicles	ER dynamics Endocytosis Phagosome maturation GLUT4 translocation	AD Upregulated in AD brains KD decreases Aß production Phosphorylated Rab10 present in NFTs	[73,74,75,76,77,78]
**Rab11A/B**	Golgi Recycling endosome	Transport through recycling endosomes Exocytosis	PD Overexpression rescues dopaminergic neuron loss and behavioural deficits HD Impaired activity in HD HTT regulates apical vesicle trafficking of PAR3-aPKC via Rab11A Rab11 dysfunction in HD leads to impaired GLUT3 trafficking contributing to glucose hypometabolism Overexpression rescues neurodegeneration locomotor defects and synaptic changes in HD Overexpression rescues synaptic vesicle defects Dominant active Rab11 normalises cysteine uptake, subsequently increasing glutathione synthesis, clearing ROS, and improving neuronal survival in HD neurons Dominant active Rab11 protects HD neurons from glutamate induced death AD Interacts with presenilins Involved in Aß trafficking	[19,20,79,80,81,82,83,84,85,86,87,88,89,90]
**Rab13**	TGN Recycling endosomes Plasma membrane	Tight junction maintenance and formation TGN and recycling endosome trafficking	PD Overexpression reduces α-synuclein toxicity	[91,92,93]
**Rab19**	Golgi	Unknown	HD Reduced Htt perturbs Rab19+ vesicle trafficking	[31,94]
**Rab27b**	Secretory vesicles Plasma membrane	Secretion and exocytosis regulation	PD Increases α-synuclein clearance via autophagy, decreasing toxicity AD Upregulated	[45,95,96,97]
**Rab29**	TGN Golgi	TGN maintenance and retrograde trafficking	PD Recruits and stimulates LRRK2	[98,99,100]
**Rab31**	TGN Golgi Endosomes	TGN vesicle formation and trafficking to the endosomes TGN to plasma membrane trafficking EGFR trafficking to late endosomes	PD Mediates transport of α-synuclein extracellular vesicles	[71,101,102,103,104]
**Rab32**	ER Mitochondria	Autophagic vacuole formation in autophagy Phagosome maturation Mitochondrial dynamics	PD Regulation of LRRK2	[62,105,106,107,108]
**Rab35**	Plasma membrane	Endocytic recycling	AD Involved in tau degradative pathway	[109,110,111]
**Rab39B**	Golgi Early endosome	ER to Golgi GluA2 AMPAR subunit trafficking Synapse formation and maintenance Autophagy	PD Loss of function Rab39B linked to early onset PD, and causes α-synuclein dysregulation AD Sequestered into Aß plaques	[69,112,113,114,115]
